# Enhancement of Contact Lens Disinfection by Combining Disinfectant with Visible Light Irradiation

**DOI:** 10.3390/ijerph17176422

**Published:** 2020-09-03

**Authors:** Katharina Hoenes, Barbara Spellerberg, Martin Hessling

**Affiliations:** 1 Institute of Medical Engineering and Mechatronics, Ulm University of Applied Sciences, Albert-Einstein-Allee 55, 89081 Ulm, Germany; martin.hessling@thu.de; 2 Institute of Medical Microbiology and Hygiene, University of Ulm, Albert-Einstein-Allee 11, 89081 Ulm, Germany; barbara.spellerberg@uniklinik-ulm.de

**Keywords:** photoinactivation, 405 nm, visible light, synergy, contact lens disinfection solution, multipurpose solution, pseudomonas

## Abstract

Multiple use contact lenses have to be disinfected overnight to reduce the risk of infections. However, several studies demonstrated that not only microorganisms are affected by the disinfectants, but also ocular epithelial cells, which come into contact via residuals at reinsertion of the lens. Visible light has been demonstrated to achieve an inactivation effect on several bacterial and fungal species. Combinations with other disinfection methods often showed better results compared to separately applied methods. We therefore investigated contact lens disinfection solutions combined with 405 nm irradiation, with the intention to reduce the disinfectant concentration of ReNu Multiplus, OptiFree Express or AOSept while maintaining adequate disinfection results due to combination benefits. Pseudomonads, staphylococci and *E. coli* were studied with disk diffusion assay, colony forming unit (cfu) determination and growth delay. A log reduction of 4.49 was achieved for *P. fluorescens* in 2 h for 40% ReNu Multiplus combined with an irradiation intensity of 20 mW/cm^2^ at 405 nm. For AOSept the combination effect was so strong that 5% of AOSept in combination with light exhibited the same result as 100% AOSept alone. Combination of disinfectants with visible violet light is therefore considered a promising approach, as a reduction of potentially toxic ingredients can be achieved.

## 1. Introduction

With approximately 125 to 140 million contact lens wearers worldwide [1,2,3] (numbers from 2004 and 2010) the prevention of lens-related infection is a serious healthcare issue. Several ocular diseases are associated with contact lens wear, such as contact lens acute red eye (CLARE), contact lens peripheral ulcer (CLPU) and infiltrative keratitis [4,5,6,7]. Due to the high numbers of contact lens users, even complications with a rare occurrence will concern a considerable number of patients.

The incidence of contact lens related microbial keratitis is 1.9 per 10,000 for daily wear of soft contact lenses in Australia [8] and 1.8–2.44 per 10,000 in Scotland for all types [9], reaching up to 3.09 per 10,000 in Hongkong [10]. Estimates of risk appear stable over time as quantified over a 20 year period [11,12]. Contact lens wearers thus have an approximately five- to seven-fold higher risk of microbial keratitis compared to non-contact lens wearers [9,10], with increasing risk for extended or overnight wear.

One of the problems might be the partially insufficient effectiveness of contact lens disinfection solutions. When testing other isolates than the given microbial test strains in the normative standard for a species, the disinfection results of commercial solutions are insufficient in some cases [13,14,15]. Nevertheless, it is not recommendable to enhance the antimicrobial impact of contact lens disinfection systems by increasing the concentration of the solutions. The reason for this is the potential toxicity to epithelial structures of some contact lens solution ingredients. It is reported [16] that the use of preserved lens care solutions led to an increased *P. aeruginosa* binding, presumably by an up-regulation of receptors on corneal epithelial cells, while at the same time a disruption in epithelial homeostasis occurred. Another study [17] found a 12-fold increase of *P. aeruginosa* uptake into the corneal epithelium of rabbits following the wear of multipurpose solution-soaked lenses. Uptake of preservatives into different types of polymeric lens materials was demonstrated [18], as well as the release of disinfectant, occurring after reinsertion of the lens to the ocular surface.

Several studies testing chemical disinfection solutions on epithelial cells demonstrated that already the limit of health compatibility has sometimes been reached. Various epithelial cell cultures showed cell membrane damage [19], loss or damage of tight junctions [19,20], altering of cell shape and size, loss of mitochondrial enzyme activity, inflammatory response [21], activation of cell death receptors [22,23] or reduced viability [21,24,25,26].

The inactivation of microorganisms by irradiation with visible light, especially in the violet and blue spectral range, has been a recent topic in disinfection research [27,28]. Endogenous photosensitizers absorb radiation of distinct wavelengths and induce the formation of reactive oxygen species (ROS), which attack microbial targets [29,30,31]. As most bacterial and fungal species harbor porphyrins and flavins, which are considered as relevant responsible photosensitizers, the sensitivity of over 40 different microbial species, including bacteria and fungi, towards visible light has been demonstrated [32,33]. Even viruses have successfully been inactivated by exposure to 405 nm in phosphate buffered saline [34] or nutrient broth [35] with doses of 2804 J/cm^2^ for a 3.9 log reduction and 510 J/cm^2^ for a 5.4 log reduction, respectively.

Previous work suggested reducing microbial burden in contact lens care by applying a light dose, destructive of relevant microorganisms and fungi [36]. This could be achieved by using transparent contact lens cases in combination with a LED-equipped base irradiating the inside from beyond [37].

As there seem to be synergistic or at least combined effects of irradiation techniques such as photodynamic therapy (PDT) and antibiotics [38,39] we investigate whether similar effects occur when combining contact lens disinfection solutions and LED-based irradiation at 405 nm. Few investigations of visible light, without the addition of external photosensitizers, in combination with other antimicrobial approaches have been performed. Fila et al. [40] examined irradiation at 405 nm in combination with antibiotics on different Pseudomonas strains by checkerboard assay without using external dyes. Another strategy was applied by Moorhead et al. [41] combining 405 nm irradiation with chlorinated disinfectants against *Clostridium difficile* spores. 460 nm led to an antibacterial effect in a triple combination together with ineffective antibiotics and non-effective silver nanoparticles [42]. Pure H_2_O_2_ combined with blue light of 450–490 nm was especially effective in two independent studies [43,44]. All of these studies noticed an increased effect of the combination compared to single methods, which were sometimes used in sub-lethal concentrations, but not all of them tested for synergy.

When examining the combination of two different techniques an analysis procedure for quantification of effectiveness has to be specified. The term “synergy” is often used, which is colloquially defined as an effect exceeding the sum of the single effects when performing both techniques simultaneously [45]. However, there is a lack of definition for this term in normative standards [45,46]. In many research works entitled with the term “synergy” there is often no detailed analysis carried out concerning this phenomenon as long as the effect of the two combined methods exhibits an enhanced impact [47,48,49]. Other studies define specific decision criteria, such as a reduction increase of 2 log for the combination compared to the most effective single component, as a definition of synergy [50].

The American Society for Microbiology conscientiously defined experimental procedures for determining synergistic effects, which are disk diffusion assays, E-tests for antibiotic susceptibility, checkerboard assays, post-antibiotic effects (PAE) and the Bliss model for biofilm testing [38]. Others claim that, because a synergism is a physiochemical mass-action law issue, it has to be calculated with Combination Index (CI) values [51], based on Loewe Additivity.

Foucquier et al. [45] deliver an overview of the mathematical background for calculations of combination effects. The authors divide approaches into effect-based and dose-effect based. “Response Additivity” is defined as the improvement when comparing the combined effect with the additive effect of both single agents, which would be the colloquial understanding of synergy. This definition belongs to the effect-based group of strategies, which inherit some limitations like, in this case, assumed linear dose-effect curves for both agents. Dose-effect-based strategies, however, rely on the mathematical framework of Loewe Additivity [52] considering non-linear dose-effect curves, determining which concentration of each drug alone produces the same effect as the combination, rather than comparing effects of given concentrations. This approach requires a certain amount of data and can rapidly become demanding. Generally, any defined effect level can be used for comparison [46]. Measurement variable can be any parameter giving knowledge about bacterial condition, such as colony forming units [38,41], change of color [53] or OD_600_ (optical density at 600 nm) values after a specified incubation [38,40,54], as terms in the equation are dimensionless quantities [46]. From the results, the Combination Index (CI), also called Fractional Inhibition Concentration (FIC), can be calculated for several concentration/dose combinations, which is considered to be the most suitable analysis for synergy testing [45].

Several slightly variant categorizations of CI values and their meanings exist. In this study, one of the earliest definitions from Chou is applied, which he later refined [46] in the categories defining synergism: slight synergism (0.85–0.9), moderate synergism (0.7–0.85), synergism (0.3–0.7) and strong synergism (0.1–0.3). CI values exceeding 1 are called nearly additive (0.9–1.10), slight antagonism (1.10–1.20), moderate antagonism (1.2–1.45), antagonism (1.45–3.3), and strong antagonism (3.3–10).

In cases of microbial keratitis associated with contact lens wear, predominantly environmental organisms were isolated as causative agents, with *P. aeruginosa* being the most frequently recovered organism [55,56,57,58]. The strong association between *P. aeruginosa* and ocular infections might also be caused by a suitable environment for Pseudomonads in the system of lens and storage cases. Microbial keratitis in contact lens wear is frequently associated with the presence of biofilm in the contact lens case [59]. Pseudomonas species are known to be biofilm builders [7] and the storage case gives a good environment for proliferation [59]. In a study of various *Pseudomonas aeruginosa* isolates some demonstrated the ability to grow to levels above the initial inoculum in one of the chemical disinfectants examined [15].

For this reason, we chose a Pseudomonas strain for most of our experiments. Since we are not allowed to cultivate pathogenic strains in our facilities, experiments were carried out with *Pseudomonas fluorescens*. In regard to visible light irradiation, it seems that relatives of the same species act similarly [32,40].

In this study we applied a disk diffusion assay, cfu (colony forming unit) determinations on agar plates and nutrient pads, including different procedures for the post-exposure elimination of the disinfection solution. For analysis on agar plates the calculation of Combination Index values based on Loewe Additivity was performed. Furthermore, the monitoring of growth delay, similar to post-antibiotic effect studies (PAE), was applied as method to investigate combination effects of contact lens disinfection solutions and visible light irradiation at 405 nm.

## 2. Materials and Methods

### 2.1. Bacterial Strains and Contact Lens Disinfection Solutions

*Pseudomonas fluorescens* (DSM4358), *E. coli* (DSM1607) and *S. carnosus* (DSM20501) were obtained from DSMZ (Deutsche Sammlung für Mikroorganismen und Zellkulturen, Braunschweig, Germany). Pseudomonads were cultivated in 535 medium (30 g tryptic soy broth (Sigma-Aldrich Chemie GmbH, München, Germany) per liter) in an overnight culture of 3 mL at 30 °C and 170 rpm. 200 µL of this pre-culture was cultivated in 30 mL fresh medium at 30 °C and 170 rpm until an optical density of 0.35 in mid-exponential phase was reached. For *E. coli* and *S. carnosus* the same procedure at 37 °C was applied with M92 medium (30 g tryptic soy broth (Sigma-Aldrich Chemie GmbH, München, Germany), 3 g yeast extract (Merck KGaA, Darmstadt, Germany) per liter) for *S. carnosus* and LB medium (10 g tryptone (VWR international, Leuven Belgium), 5 g yeast extract (Merck KGaA, Darmstadt, Germany), 10 g sodium chloride (VWR international, Leuven Belgium) per liter) for *E. coli*. Bacterial cultures were centrifuged at 7000× *g* for 5 min and the resultant pellet resuspended in phosphate buffered saline (PBS). After a further washing step in PBS the suspension was diluted to the desired population density for experimental use in PBS.

For disk diffusion assays the bacterial solution was diluted to 0.5 McFarland standard, which was approximately 10^8^ CFU/mL. Instead of Müller-Hinton-Broth commonly applied for this assay type, 535 medium was used and poured in equally filled dishes with 10 mL per 90 mm diameter dish. For nutrient pad analysis, the solution was adjusted to 6–8 × 10^7^ CFU/mL, as the detection limit for the reduction lies one log beyond the used starting concentration and an approximately 6 log reduction was pre-determined for 100% ReNu Multiplus combined with light. For agar plate assays a concentration of 5 × 10^5^ to 10^6^ CFU/mL was adjusted, referring to the recommendation of the normative standard for contact lens solution testing [60]. Likewise, samples for growth delay analysis were adjusted to a concentration of 5 × 10^5^ to 10^6^ CFU/mL for the irradiation/disinfection solution exposure treatment. As medium was added for incubation in the microplate reader in a proportion of 1:10, the final concentration for incubation was diluted by one log. The bacterial concentrations indicated represent the concentration in the well already mixed with different concentrations of contact lens solutions. Bacteria were plated on the same media as applied in the fluid culture. Dey-Engley neutralization broth (DEB, Thermo Fisher Scientific, Waltham, MA, USA) was used to eliminate the effect of disinfection solutions after treatment for agar plate and growth delay assays. For nutrient pad assays pseudomonads were incubated on cetrimide pads 14075–47-N (Sartorius, Göttingen, Germany) after membrane filtration.

Untreated controls were analyzed for each assay type to exclude unintended bacterial reduction by environmental factors. In cases where log reductions of sample results had the same algebraic sign as the control, the absolute value of the control was subtracted, otherwise it was ignored. By this means, reductions caused by environmental factors were taken into account, in a manner not to improve inactivation results.

Contact lens disinfection solutions examined in this study were ReNu Multiplus (Bausch+Lomb, Rochester, NY, USA), OptiFree Express (Alcon, Fort Worth, TX, USA) and AOSept Plus (Alcon, Fort Worth, TX, USA). All solutions were used within expiration date.

### 2.2. Irradiation Setup

For irradiation a LED light source of 405 nm was applied (LZ4–40UB00–00U8 (LED Engin, Inc., San Jose, CA, USA). The emission was measured with a spectrometer (SensLine AvaSpec-2048 XL, Avantes, Appelsdorn, The Netherlands), after a pre-heating interval. The measured peak emission was determined at 405.9 nm with a bandwidth of 19 nm. The LED was mounted to a heat sink, which was actively cooled with a fan during experiments to avoid heating the sample. This package was placed on top of a truncated hollow pyramid with a high reflective inside, which ensured that the sample area was irradiated homogenously (described earlier in [61]). Experiments were performed in 48 well plates placed on a black underground to avoid unintentional potentiation of irradiation by light reflection from the white laboratory table. 1 mL of sample was transferred into several wells of a 48 well microtiter plate and the pyramid placed on top of the plate, covering 3 × 5 wells. The average sample temperature measured with an infrared thermometer (Raytek Fluke Process Instruments GmbH, Berlin, Germany) was 23.8 °C, with a maximum of 26.2 °C. Irradiation intensity depended on the experimental series and was adjusted by means of an optical power meter OPM150 (Qioptiq, Göttingen, Germany).

### 2.3. Disk Diffusion Assay

Disk diffusion assays are a technique anchored in routine clinical microbiology, especially in antibiotic susceptibility testing. The measurement parameter is the formation of circular growth inhibition zones, which are caused by diffusion of the applied drug from impregnated disks through the agar medium. No detailed definition of synergy is given for this method in official guidelines, although Wozniak et al. [38] defined synergy in a disk diffusion assay as an increase of the inhibition zone by 2 mm in combined treatment compared to the single treatment values.

Dilutions of the examined contact lens disinfection solutions in PBS were prepared directly before use to concentrations of 100, 80, 60, 40, 20 and 5%, respectively. 100% refers to the formulation of the specific disinfection solution that is commercially available. Bacterial solutions were irradiated as described above and plated on 535 agar plates of defined thickness. Irradiation doses used for disk diffusion assays have been 0 J/cm^2^ as control, and 35 J/cm^2^, 70 J/cm^2^ and 140 J/cm^2^, achieved in different time intervals with an intensity of 20 mW/cm^2^. For the plating technique a volume of 1 mL was distributed by rotary movement of the dish, letting plates air dry afterwards. As the large volume would increase the applied bacterial concentration designed for a 100 µL application, the suspensions were diluted in PBS by 1 log before plating. Soaked disks were placed manually with flamed forceps. After incubation for 24 h at 30 °C, inhibition zones were determined manually by fitting circles to a photograph of the plates in an image processing program. All plates were prepared in duplicates and each experiment was repeated three times. *P. fluorescens* and all three contact lens solution types were investigated in this assay.

### 2.4. Determination of Bacterial Reduction with Nutrient Pads

Determinations of cfu were performed on *P. fluorescens* for combinations of ReNu Multiplus multipurpose solution and 405 nm visible light at a dose of 140 J/cm^2^. This dose was chosen as it is easily reachable within an overnight disinfection, even when considering a low-cost LED as a potential irradiation product instead of the high-power LED used in the test setup. On the other hand, this dose exhibits a moderate effect when applied alone so that a combination treatment will still result in bacterial concentrations above the detection limit. Concentrations of 5, 20, 40, 60, 80 and 100% of ReNu Multiplus were tested on *P. fluorescens* as single treatment and in combination with 405 nm irradiation at 20 mW/cm^2^ in a time interval of 2 h, as well as the effect of light alone in PBS (0% ReNu Multiplus). The bacterial starting concentration was 6–8 × 10^7^ CFU/mL. 100 µL sample volume was diluted serially in PBS. A volume of 500 µL of the desired dilution was then immediately subjected to membrane filtration to eliminate the disinfection solution. Bacteria remained on the filters with a pore size of 0.45 µm, which were placed on moistened nutrient pads. After incubation at 30 °C for 30 h, disks were photographed and colonies enumerated manually. The resultant count was converted to CFU/mL, and in log reduction referring to the plated starting concentration. Each experiment was performed in triplicates and repeated three times.

### 2.5. Determination of Bacterial Reduction with Agar Plates

Just as for cfu determinations on nutrient pads, an irradiation dose of 140 J/cm^2^ was chosen. The bacterial starting concentration was 5 × 10^5^ to 10^6^ CFU/mL, as recommended in the normative standard for contact lens solution testing. In this test series three different irradiation intensities were selected to reach this dose within different time intervals. With 10, 20 and 40 mW/cm^2^ the defined dose was reached within 4, 2 and 1 h irradiation time respectively. This will automatically lead to different residence times for the disinfection solution, whereas 4 h is the minimum disinfection time given by the contact lens solution manufacturer. Each experiment for the combination effect was performed in triplicate and repeated three times.

To be able to calculate the CI value, reference experiments for the disinfection procedures applied separately were carried out in triplicates and repeated twice. Irradiations with 405 nm at 10, 20 and 40 mW/cm^2^ on bacteria in PBS as well as the effect of ReNu Multiplus without irradiation over intervals of 4, 2 and 1 h at concentrations of 0, 5, 20, 30, 40, 50, 70, 80 and 100% serve as reference for the combined experiments.

100 µL of each sample was transferred to 900 µL Dey-Engley neutralizing broth (DEB) and incubated for at least 15 min at room temperature. DEB samples were diluted to proper bacterial concentrations in PBS and plated manually with a glass spatula. After incubation for 30 h at 30 °C agar plates were photographed and enumerated manually. The resultant count was converted to CFU/mL, and in log reduction referring to the plated starting concentration. Each experiment was performed in triplicate and repeated at least three times.

Based on Loewe Additivity, CI values are then calculated as follows:

CI = a/A + b/B,(1)

where a and b are the concentrations of each agent used in the combination, while A and B are the concentrations of the agents that are necessary to reach the same effect when used separately.

Combination Indexes are generally reported without any assessment of the degree of certainty [45], but as investigations of biological systems inevitably contain experimental errors, we used the definition from Chou [46] in a conservative way and only categorized results of “moderate synergism” or more as enhanced outcome.

### 2.6. Determination of Bacterial Reduction via Regrowth Behavior

In antibiotic testing, where combined testing is frequently performed, post antibiotic effects (PAE) indicate the delay of the regrowth after the exposure to a drug over a certain period and can likewise be used to monitor the differences between single drugs and their combination. The difference to checkerboard assays is that the exposure time is limited, and the drug is removed or eliminated thereafter. As continuous irradiation is not possible inside a microplate reader during incubation, the effect of the disinfection solution equally has to be stopped to achieve comparable results. As this scenario would also represent a realistic application for contact lens care, this method was chosen in place of a checkerboard assay in this study.

The exposure time was set to 4 h as this is the smallest time interval given in manufacturer instructions for contact lens disinfection solutions. This leads to an irradiation intensity of 10 mW/cm^2^ to reach a dose of 140 J/cm^2^. Furthermore, higher irradiation intensities of 20 and 40 mW/cm^2^ were tested with exposure times of 2 and 1 h, respectively. Contact lens disinfection solution concentrations were tested at 40, 30, 20 and 5% of the commercially available formulation. Besides ReNu Multiplus, another multipurpose solution was examined against *P. fluorescens*. OptiFree Express has often been reported to achieve high bacterial impact, but at the same time is aggressive to human ocular epithelium [62]. Therefore, it would be desirable to reduce concentration of ingredients through a combined use with light. Besides another multipurpose solution, further strains (*S. carnosus* and *E. coli*) were tested with this technique together with ReNu Multiplus and visible light. 

After exposure, samples of 100 µL were immediately transferred to 900 µL of DEB to neutralize the effect of the disinfection solution. This was also performed with samples that have only been irradiated in PBS. After incubation for at least 15 min at room temperature 20 µL of each sample was transferred into a 96 well plate and mixed with 180 µL of specific growth medium. The violet color of DEB thereby was diluted by factor 1:10 so that the sample was translucent enough to monitor increasing turbidity through growth in a microplate reader. Microtiter plates were incubated in a Clariostar Plus (BMG Labtech, Ortenberg, Germany) at 30 °C for *P. fluorescens* and at 37 °C for all other strains for at least 30 h with measurement of OD_600_ in 5 min intervals and shaking for 30 s before each measurement, ensuring almost continuous rotary growth conditions. Additionally, sequential ten-fold dilutions of each strain in untreated condition were measured with the same protocol. Each experiment was repeated three times. Depending on how many bacteria were inactivated during exposure of light and/or disinfection solution, the regrowth will be delayed. Based on the untreated dilutions, a calibration curve could be prepared, putting into context the measured OD value at a certain time towards the underlying log reduction.

## 3. Results

### 3.1. Disk Diffusion Assay

**Figure 1:**
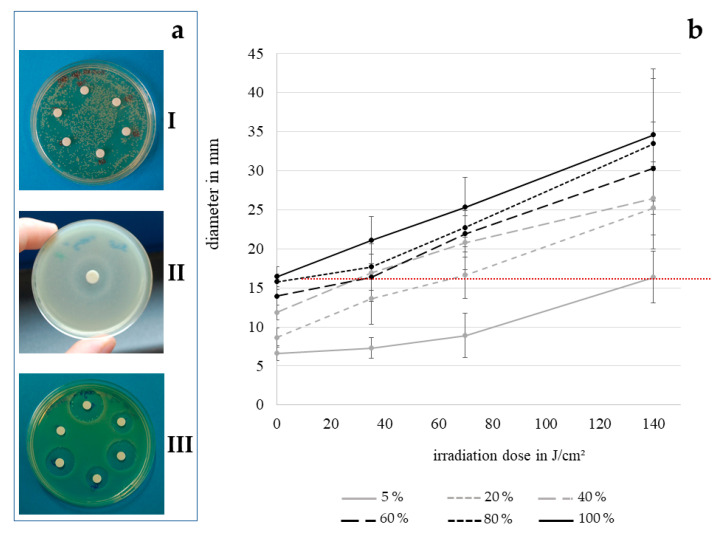
Demonstration of different agar plate appearances (**a**) with fragmentary lawn through excessive light dose of 280 J/cm^2^ (**I**), indefinite inhibition zone with 130 mg/10 mL agar concentration and 100% ReNu Multiplus (**II**), optimal appearance with different concentrations of AOSept (**III**). Diameter of inhibition zones on *P. fluorescens* lawn achieved with different concentrations of hydrogen peroxide solution AOSept, dependent on the dose of 405 nm applied with 20 mW/cm^2^ (**b**). Error bars indicate the deviation in the three experiments. The red dotted line indicates the size of inhibition zones generated with 100% AOSept without irradiation.

To assess the antibacterial effect of contact lens disinfectant solution by disk diffusion assay, we applied ReNu Multiplus and OptiFree Express at concentrations of up to 100% to the agar plates. However, the multipurpose solutions used for this study did not form clear inhibition zones in any concentration that was tested. As we assumed this fact to be caused by the molecular structure of the active components, not being able to pass the cross-linked agar, we tried to decrease the agar concentration in the plates until the limit of solidity in order to achieve greater pore sizes. However, with 110 mg/10 mL agar still no enhanced effect on the appearance of inhibition zones formed by the multipurpose solutions alone or in combination with visible light was identifiable, even at 100%. With unclear inhibition zones, only visible with background lighting (Figure 1aII), disk diffusion results with multipurpose solutions were considered not analyzable. Cfu determinations of contact lens disinfection solution, however, showed a considerable decrease in bacterial count. With the hydrogen peroxide based solution AOSept, conversely clearly visible inhibition zones were detectable (Figure 1aIII).

As can be seen in Figure 1aI, the irradiation dose has to be selected carefully for this technique, as otherwise a semiconfluent growth of colonies, which is required for the development of clearly visible inhibition zones, cannot be achieved. The disinfection effect increases with the irradiation dose as shown in Figure 1b. The highest dose analyzable with a continuous bacterial lawn was 140 J/cm^2^ at 405 nm. Likewise, the inhibition zones increase with the percentage of hydrogen peroxide solution. The dotted line on the graph represents the disinfection result when using the disinfectant at 100% concentration, as commercially available. Every data point above this line, leading to greater inhibition zones, shows the benefit of combining conventional contact lens disinfection techniques with visible light irradiation. The combination of 140 J/cm^2^ irradiation at 405 nm with 5% of the original concentration of the disinfection solution achieves a similar result as a 100% solution without irradiation. Any higher concentrations leads to even better results in combination with 405 nm.

### 3.2. Determination of Bacterial Reduction with Nutrient Pads

**Figure 2:**
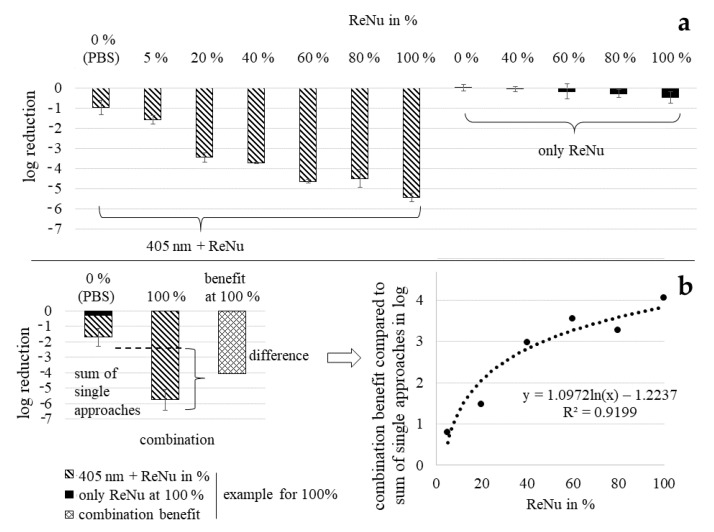
Reduction results with nutrient pads for all combinations tested including single approaches as reference. Error bars indicate the deviation in the three experiments (**a**), Example of “synergy“ calculation as enhancement of the combination over the sum of ReNu Multiplus and light applied separately, and progress of these “synergies“ for different ReNu Multiplus concentrations on *P. fluorescens* (**b**).

Testing disinfection solution ReNu Multiplus as single treatment with nutrient pads, nearly no inactivation was observable (Figure 2a). For 100%, which represents the pure commercially available solution, only 0.48 log reduction was achieved in 2 h at 20 mW/cm^2^ with a bacterial starting concentration of 6–8 × 10^7^ CFU/mL. In contrast, a combination treatment with ReNu Multiplus 100% and visible light of 405 nm was quite successful with almost complete inactivation of the bacteria. Even at lower disinfectant concentrations, considerable bacterial reduction was achieved. Plotting the benefit of combination treatment compared to the sum of single approaches, colloquially called synergy (Figure 2b), it becomes visible that the relation is not linear. The higher the applied disinfection solution concentration, the greater is the overall combined bacterial inactivation. However, the increase of the benefit slows down for higher concentrations. The most distinct difference in the gradient of achieved log reductions lies between 20 and 40% ReNu Multiplus content with 2.86 and 4.38 log, respectively. Therefore 40% of ReNu Multiplus in combination with visible light of 405 nm appears to be the best compromise between reducing the disinfectant concentration and achieving optimal inactivation results. All other experiments with different methods were therefore carried out with disinfectant concentrations in this range. Light irradiation with 405 nm alone led to 1.42 log reduction.

With nutrient pad analysis we determined that any combination and even light alone produces better results in a 2 h treatment than application of ReNu Multiplus at 100% for a *P. fluorescens* concentration of 6–8 × 10^7^ CFU/mL.

### 3.3. Determination of Bacterial Reduction with Agar Plates

**Figure 3:**
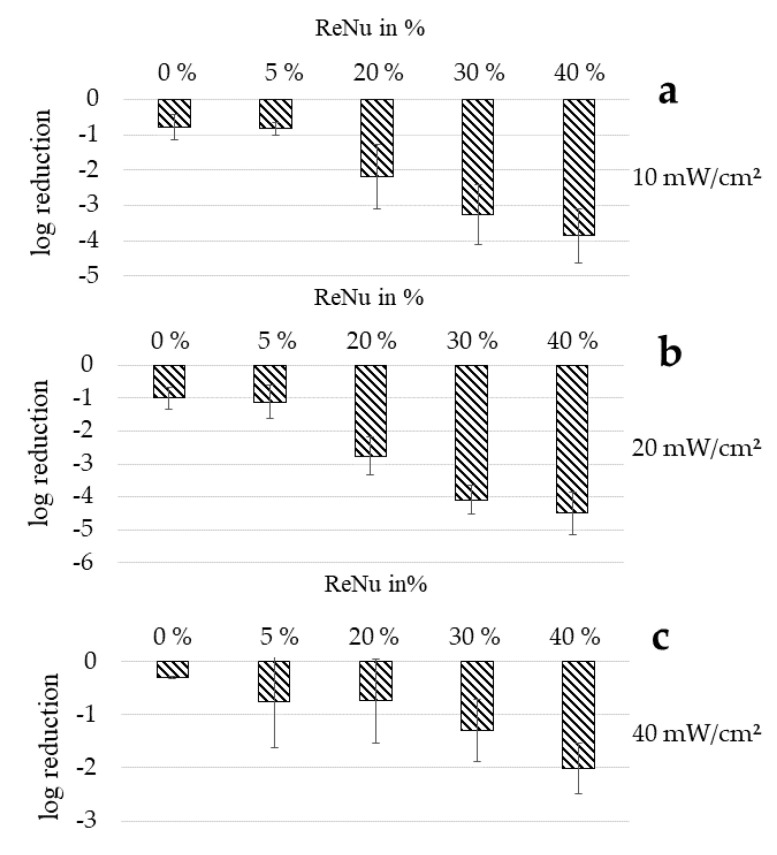
Log reduction results for combinations of different concentrations of ReNu Multiplus with visible light of 405 nm on agar plates. A dose of 140 J/cm^2^ was reached with 10 mW/cm^2^ in 4 h (**a**), 20 mW/cm^2^ in 2 h (**b**), 40 mW/cm^2^ in 1 h (**c**). Error bars indicate the deviation in the three experiments.

To determine if the results of the nutrient pad analysis can be confirmed with other methods, agar plate determinations of bacterial inactivation were carried out (Figure 3). In Figure 3, the inactivation effect tested with cfu determinations on agar plates is depicted for different irradiation intensities. The bacterial starting concentration was 5 × 10^5^ to 10^6^ CFU/mL. Comparing the different investigations methods, the combination results correspond relatively well. For a 20 mW/cm^2^ irradiation in PBS without disinfectant a 1.42 log decrease of bacterial counts is achieved on nutrient pads (Figure 2a) while 1.00 log is measured with agar plates (Figure 3b). For a combination of 405 nm irradiation with 5, 20 and 40% of ReNu Multiplus, 2.16, 2.86 and 4.38 log decreases are achieved on nutrient pads, while the results on agar plates are 1.12, 2.76 and 4.49 log, respectively.

Comparing different irradiation intensities reaching the same dose of 140 J/cm^2^ over various exposure times it becomes evident that the highest irradiation intensity is not necessarily the best choice. At 40 mW/cm^2^ for 1 h (140 J/cm^2^) the lowest reduction results are achieved with only a 2.01 log decrease at 40% ReNu Multiplus in combination with 405 nm irradiation. Best combination results were achieved at 20 mW/cm^2^ and 40% concentration of ReNu Multiplus with 4.49 log reduction.

### 3.4. Loewe Additivity

**Figure 4:**
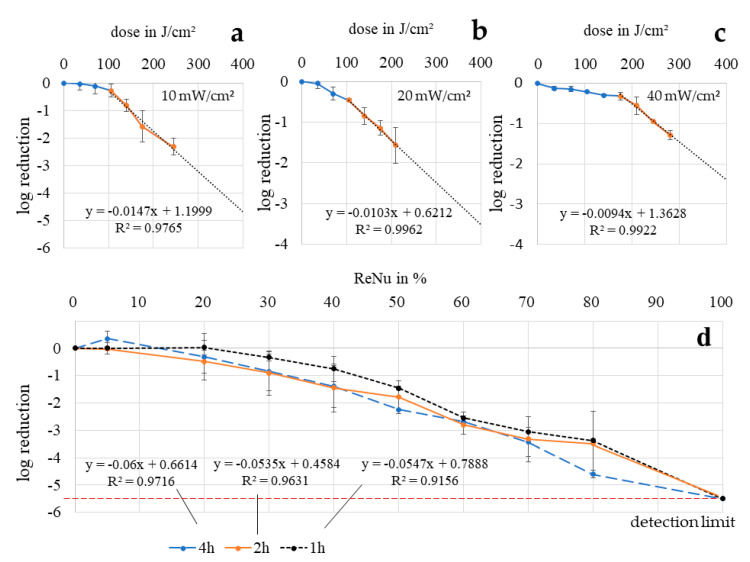
Reference experiments on agar plates to achieve dose-effect curves for single approaches on *P. fluorescens*: 405 nm irradiation in phosphate buffered saline (PBS) with 10 mW/cm^2^ (**a**), 20 mW/cm^2^ (**b**) and 40 mW/cm^2^ (**c**) as well as ReNu Multiplus exposure for 1, 2, and 4 h at different concentrations (**d**). Error bars indicate the deviation in the two experiments.

For comparing the combination effects with the single approach results, ReNu Multiplus and 405 nm alone have not only been tested at analogous concentrations/doses as the combinations, but over the whole concentration range and over an extended dose range (Figure 4). This allows the calculation of CI values based on Loewe Additivity, which directly gives a benchmark for synergy categorization. Interestingly, there was no large difference between exposure times of 1, 2 and 4 h for ReNu Multiplus at any concentration (Figure 4d). There is a tendency towards better efficacy for longer duration, but the deviation is lower than expected. Only at 80% disinfection solution did the 4 h results exceed the results of shorter durations, with a decrease of 4.60 log compared to 3.49 and 3.38 log at 2 and 1 h, respectively. At 100% ReNu Multiplus there were no colonies visible after any exposure time starting from a concentration of 5 × 10^5^ to 10^6^ CFU/mL. Uniform behavior over disinfection solution concentration was assumed here, represented by a linear fit. For references at 405 nm irradiation, bacterial solutions in PBS were irradiated with 10, 20 and 40 mW/cm^2^ over different intervals reaching a dose of 245 J/cm^2^. The typical behavior for visible light photoinactivation, with a mostly linear slope in a half logarithmic representation and an additional shoulder at the beginning (non-mono-exponential), becomes evident here. Four data points within the linear section were achieved for each irradiation intensity. A linear fit through those points was used as reference for 405 nm irradiation as a single approach to calculate the doses necessary to reach a certain effect.

In Table 1 the calculated CI values are presented, based on the combination results shown in Figure 3 and the trendlines from Figure 4, calculated with the Formula (1).

For 20 mW/cm^2^ and 2 h irradiation all values lie considerably below 0.85 indicating moderate synergism for 20 and 40% and even synergism for 30%. At this irradiation intensity, the absolute log reductions also reach the highest values with 4.09 log at 30% and 4.49 log at 40%. It is noteworthy that at 10 mW/cm^2^ as well as 20 mW/cm^2^ the CI values for 30% ReNu Multiplus content are the lowest, showing the best beneficial effect for the combination. This fits with the decrease of the slope for higher concentration values and the leap between 20 and 40% noticed at the nutrient pad results (Figure 2b). None of the combinations examined achieves better results than the samples for ReNu Multiplus at 100% - at least in the test series with agar plates and low bacterial concentrations - as none of the results in column B shows a percentage over 100%. However, good log results were achieved at 30 and 40% for 10 mW/cm^2^ and 20 mW/cm^2^. Regardless, CI values for 10 mW/cm^2^ and also for 40mW/cm^2^ lie close to or even above 1, indicating no synergism.

Nevertheless, such a combination approach could be employed in practice regarding overall reductions and taking into account that they have been achieved with clearly lower concentrations. According to the testing on agar plates the overall result cannot be improved, but the concentration of antibacterial ingredients in the formulation of contact lens solutions could be reduced when combining with light to preserve the consumer’s epithelial health, while still achieving acceptable results.

### 3.5. Effectiveness Dependency of Multipurpose Solution ReNu Multiplus on Bacterial Concentration

**Figure 5:**
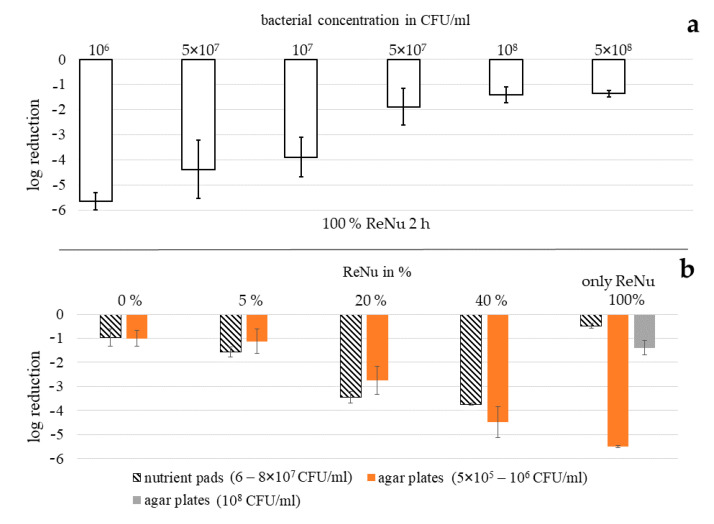
Effectiveness of undiluted ReNu Multiplus disinfection solution (100%) against *P. fluorescens* at different bacterial concentrations, tested with agar plates. Error bars indicate the deviation in the three experiments (**a**). Comparison of log results with nutrient pads and agar plates for combination of ReNu Multiplus and visible 405 nm light at 20 mW/cm^2^ for 140 J/cm^2^. Error bars indicate the deviation in the three experiments (**b**).

Comparing the inactivation results for ReNu Multiplus as a single method achieved with agar plates (Figure 4d) and nutrient pads (Figure 2a), great differences were observed. While no colonies were visible after 2 h of exposure to 100% ReNu Multiplus and subsequent distribution on agar plates (5.5 log reduction), only a 0.48 log reduction was reached with membrane filtration and nutrient pads. A major difference in the two assays was the bacterial starting concentration with 5 × 10^5^ to 10^6^ CFU/mL for the agar plate assay and 6–8 × 10^7^ CFU/mL for the nutrient pad analysis. We therefore assumed that the effectiveness of ReNu Multiplus was highly dependent on the bacterial concentration. Testing this hypothesis, we could show that the log reduction decreases with increased bacterial concentration (Figure 5a). For a 2 h exposure with a 10^6^ CFU/mL starting concentration, again no CFU were observable. Yet, at 5 × 10^7^ CFU/mL only 1.9 log reduction, and at 10^8^ CFU/mL only 1.4 log reduction, were achieved respectively, referring to the specific starting concentration.

In Figure 5b the log results for the combination approach with visible light are presented in direct comparison between agar plate and nutrient pad assay. At samples where 405 nm was additionally applied to the different ReNu Multiplus concentrations, the differences in the bacterial inoculum do not seem to play a pronounced role. This is contrary to the disinfection results with 100% ReNu Multiplus as single approach, where an increased bacterial load leads to noteworthy loss in effectiveness.

### 3.6. Determination of Bacterial Reduction via Regrowth Behavior

**Figure 6:**
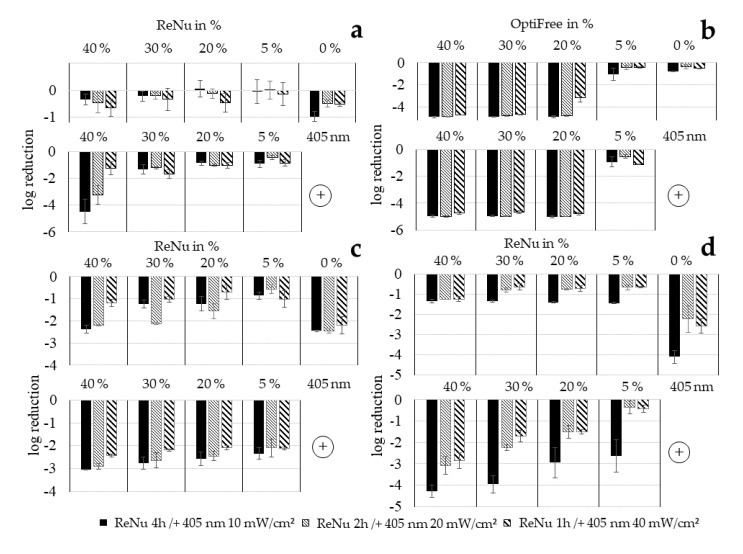
Log reductions determined from growth delay analysis for different intensities of 405 nm light and different concentrations of disinfection solutions: ReNu Multiplus on *P. fluorescens* (**a**), OptiFree Express on *P. fluorescens* (**b**), ReNu Multiplus on *E. coli* (**c**) and ReNu Multiplus on *S. carnosus* (**d**). Upper part of diagram presenting results for single approaches, lower part presenting combination results (+). Error bars indicate the deviation in the three experiments.

In this experimental series the growth of bacteria after treatment with single or combined disinfection approaches is investigated. In the literature differences in behaviour towards disinfection techniques are often detected between plated results and analytical methods in fluids. The effect of ReNu Multiplus as a single approach against *P. fluorescens* (Figure 6a) does not show a high impact and at 5% and 20% growth can occur instead of a reduction, which was already noticeable in the two other analysis methods. Log reductions at 40%, the highest ReNu Multiplus concentration tested here, varied between 0.35 and 0.64, depending on the exposure time. Compared to the results achieved with agar plates with 0.75 (1 h), 1.45 (2 h) and 1.39 (4 h) log reduction at 40%, and those of 0.0 log achieved with nutrient pads in 2 h at 40%, values reached with growth analysis lie in the middle. For irradiation with 405 nm in PBS (0%), where 0.52 (1 h/40 mW/cm^2^), 0.48 (2 h/20 mW/cm^2^) and 0.97 (4 h/10 mW/cm^2^) log were measured by growth delay, the values with agar plates reaching 0.30 (1 h/40 mW/cm^2^), 0.85 (2 h/20 mW/cm^2^) and 0.82 (4 h/10 mW/cm^2^) log reduction fit acceptable. Here the nutrient pad value with 1.42 log (2 h/20 mW/cm^2^) deviates upwards.

For combination results a huge increase occurred between 30 to 40% disinfectant concentration, at least for the experiments at 4 and 2 h duration, i.e., 10 and 20 mW/cm^2^ irradiation intensity. This was already noticed in the nutrient pad analysis. Log reductions for 40% ReNu Multiplus combined with light determined by growth analysis and agar plates are 4.48 and 3.86, respectively, for 10 mW/cm^2^ and 3.24, 4.49, as well as 4.38 for 20 mW/cm^2^ at growth, agar plate and nutrient pad analysis. For concentrations of 30% and 20% ReNu Multiplus, log reduction values determined with growth analysis are considerably lower than values achieved with the two other methods. Nutrient pad and agar plate results, however, match well at 20% with a 2.76 log reduction (2 h/20 mW/cm^2^) on agar plates and a 2.86 log reduction (2h/20 mW/cm^2^) with nutrient pads. It seems that at disinfectant concentrations below 40% plating techniques deliver more optimistic results than analysis in fluid.

In the experimental series of growth delay analysis, another multipurpose solution besides ReNu Multiplus was tested (OptiFree Express) which is known to be rather aggressive to bacteria as well as to the ocular surface. Results are shown in Figure 6b, where it stands out that, down to 20% of OptiFree Express, all bacteria were killed with the solution alone, with the exception of 1 h exposure with 20%, where 3.14 log reduction was measured. At 5%, however, only reductions lower than one log were achieved. The combination with light does not seem to have any influence on the results, neither positive nor negative.

For *E. coli* (Figure 6c) the inactivation effect of ReNu Multiplus is comparably stronger than for *P. fluorescens*. The same applies for irradiation with 405 nm. In each case, the combination approach achieves higher log reductions for *E. coli* than the specific concentration of ReNu Multiplus alone. Comparing the combination with 405 nm alone, 40%, 30% and 20% show better or equal results. The overall results for the combination of ReNu Multiplus and 405 nm investigated in the growth analysis are lowest for *E. coli* compared to *P. fluorescens* and *S. carnosus*. Nevertheless, with only 40% of ReNu Multiplus, 3.05 log can be achieved when combined with 405 nm at 10 mW/cm^2^ for 4 h.

For *S. carnosus* (Figure 6d) ReNu Multiplus as a single approach achieves better results than for *P. fluorescens* but less inactivation than for *E. coli*. The impact of light, however, was comparable with the *E. coli* results, however, for 10 mW/cm^2^ even 4.11 log was reached. Similarly, in the combination approach, it appears that the difference between the irradiation intensity of 10 mW/cm^2^ and the other intensities is clearly more pronounced, as it is in experiments with *P. fluorescens* or *E. coli*. At the same time, it has to be noted that error bars are comparably large here. There is no indication of synergistic behaviour that exceeds the sum of single approaches. Apart from 5% for 1 and 2 h, the combination clearly exhibits a stronger effect than ReNu Multiplus alone. Compared with light alone, however, there is only a slight increase of 0.86 log at 20 mW/cm^2^/2 h with 40% in the combination, while most results are similar to light irradiation alone and sometimes even reach fewer log reductions. The combination with ReNu Multiplus does not seem to lead to an increased outcome compared to an irradiation in PBS for *S. carnosus* tested with growth delay, but the results of either light approach achieve better results than ReNu Multiplus alone at the concentrations examined.

Altogether, the growth analysis seems to indicate that lower irradiation intensities at longer exposure times are more effective in the combination approach than higher irradiation intensities at shorter durations. For ReNu Multiplus as a single method, *Pseudomonas sp.* seem to be the microorganism most difficult to inactivate, which matches literature data [53] and was the reason for choosing it as principal strain in this study. The addition of irradiation increases the effectiveness of ReNu Multiplus. At a 40% concentration at least 3 log reduction could be achieved for all bacterial species tested with the combination approach. 

## 4. Discussion

In this study we tested the combination of contact lens disinfection solutions with visible violet light of 405 nm. Combining different approaches has a long history not only for disinfection techniques, but also for medical therapies. For just as long, experts have been discussing how to quantify these results. Dose-effect-based strategies seem advantageous as is explained in detail in [45], because they do not have limitations through assumptions such as linearity. Furthermore, it is recommended to use several different methods to come to a conclusion. In our investigations, we often achieved varying results for the same parameters, when testing with different analytical methods. Nevertheless, related tendencies are obvious in all test methods.

The combination effect is assumed to increase with light dose. This was observed at disk diffusion testing. With all other test methods, a fixed dose of irradiation (140 J/cm^2^) was used. Synergism of pure H_2_O_2_ combined with blue light of 470 nm has previously been reported in *S. aureus* [44]. Unfortunately, ReNu Multiplus and OptiFree Express solutions did not form clear inhibition zones on agar plates even with reduced agar concentrations. A positive combination effect could be observed for the hydrogen peroxide solution AOSept, while it is only a presumption that this would also be valid for multipurpose solutions. As high concentrations of bacteria (approximately 10^8^ CFU/mL) are applied for disk diffusion assays to produce a dense bacterial lawn, bacterial concentration dependency of multipurpose solutions, as observed in Figure 5 for colony counts, could be the reason for the absence of clearly visible inhibition zones.

As the effect of light irradiation alone increases with the dose [31,40,63,64,65], a similar dose dependency for a combined application appears likely. However, an important fact in combination testing is that it is not possible to predict the results, as some drugs have several targets or independent antimicrobial mechanisms [51]. A combination of photodynamic therapy and various antibiotics, for example, showed a decrease in development of resistance for some drugs while for other antibiotics resistance was acquired through the combination with PDT [66].

Therefore, any combination of two methods has to be investigated separately and it is not possible to test for general statements about synergy [46]. Combinations at varying doses/concentration levels can lead to very different results with the same two approaches in combination [39]. Similarly, in Table 1, where CI values are determined, 20 mW/cm^2^ and 30% ReNu Multiplus lead to explicit synergy with a CI of 0.66, while 10 mW/cm^2^ at the same concentration of ReNu Multiplus is only additive with a CI of 0.92. At a light intensity of 40 mW/cm^2^ even moderate antagonism with a CI of 1.28 occurs. At the same time, it is important to clarify that for practical considerations it is not the most important issue to attain mechanistic synergy, but to achieve a high antibacterial impact. The occurrence of synergy does not necessarily arrive at the best overall results, because the highest antimicrobial effect can occur in the absence of synergy. For practical considerations, this shows that synergy is not necessarily relevant for product design, where overall reduction is the relevant measurand, while the best synergistic grade is achieved at the highest increase of the combination’s benefit.

Concerning the irradiation intensity in combination with the multipurpose solution ReNu Multiplus, our results indicate that lower intensities used over a longer exposure period will lead to higher inactivation results, than higher irradiation intensities at shorter durations reaching the same dose. The results achieved with agar plates show that 40 mW/cm^2^ irradiation does not compete with the inactivation effect of 10 or 20 mW/cm^2^ (Figure 3) in combination with ReNu Multiplus. The same tendencies are observable at growth delay analysis for *P. fluorescens*, *E. coli* and *S. carnosus*. Combining this knowledge with the assumption of an increasing effect at higher doses, applications with long irradiation intervals seem to be advantageous.

OptiFree Express, another multipurpose solution with a very potent antibacterial effect, but likewise unhealthy for the consumer [62], shows a totally different reaction pattern than ReNu Multiplus. The solution, which kills all bacteria at a concentration of just 20%, rapidly loses activity at a concentration of 5%, while the effect of ReNu Multiplus decreases continuously with gradual dilution. For OptiFree Express the addition of light does not seem to improve effectiveness. An opposite effect can be observed for ReNu Multiplus against *S. carnosus* (Figure 6d) where the irradiation with light delivers the main impact. Only for 40% at 20 and 40 mW/cm^2^ does the addition of ReNu Multiplus lead to an increase higher than the effect of 405 nm alone. It is therefore recommended to further investigate other contact lens disinfection solutions.

A noticeable fact in this study is the huge differences of ReNu Multiplus effectiveness examined with nutrient pads compared to agar plates. The effectiveness of multipurpose solutions under different experimental conditions may depend on the bacterial inoculum. The normative standard for testing contact lens solutions [60] suggests a starting concentration between 10^5^ and 10^6^ CFU/mL. The nutrient pad experiments, however, were carried out with an inoculum of 6–8 × 10^7^ CFU/mL. In fact, the log reduction considerably decreased with rising bacterial load (Figure 5a) when testing ReNu Multiplus as a single method. This could lead to severe clinical problems as total viable bacterial counts between 10^6^ and 10^8^/mL were found in 13 out of 18 contact lens cases of patients with corneal infiltrative infections using multipurpose solutions [67]. Testing the combination of ReNu Multiplus with 405 nm irradiation, the bacterial concentration did not seem to play a pronounced role. Irradiation procedures with visible light are less dependent on the bacterial inoculum as they are based on endogenous photosensitizers, which increase in parallel to the bacterial concentration. Only absorption and scattering issues seem to limit the effectiveness for high bacterial concentrations [31]. It is remarkable that in spite of the marginal single impact of ReNu Multiplus at high bacterial loads the combination effect in the presence of ReNu Multiplus increases to values well exceeding the effect of light alone.

Concerning the plausibility of the investigation of a non-pathogenic surrogate, we evaluated the literature data available for photoinactivation of Pseudomonads. *P. aeruginosa* strains are among the most often examined microorganisms regarding visible light inactivation [33], but results for other representatives of the genus are scarce. Applying 400 nm at a dose of 100 J/cm^2^ on *P. fluorescens,* Angarano et al. [68] achieved a 0.5 log reduction, which is in good accordance with our results.

Maclean et al. [31] achieved a 1 log reduction of *P. aeruginosa* (NCTC 9009) at a dose of 42.9 J/cm^2^ with 405 nm of 10 mW/cm^2^ irradiation intensity. Fila et al. [40] examined a broad range of *P. aeruginosa* strains including wild-type strains, drug-sensitive clinical isolates and multi-drug-resistant clinical isolates with very similar behaviors of 7 log reduction at around 50 J/cm^2^. Dependent on whether the average dose is considered or if the shoulder is taken into account, this leads to a result of 7–12 J/cm^2^ for 1 log reduction. Gupta et al. [69] isolated a *P. aeruginosa* strain from patients with arthroplasties for which an averaged dose of 133 J/cm^2^ was needed for a 1 log reduction of 405 nm at 123 J/cm^2^.

To our knowledge, these are the most extreme examples showing the upper and lower values for *P. aeruginosa* eradication measured to date, the variation probably caused by differences in the setup or test protocol, as different strains examined with the same protocol react similarly [40]. Still, with 154 J/cm^2^ for 1 log reduction of *P. fluorescens* at 20 mW/cm^2,^ our results are overshooting those values. It seems that *P. fluroescens* is less susceptible to 405 nm than its pathogenic relative. 

The choice of an appropriate surrogate concerning the performance of a disinfection method should rather be more conservative and survive longer than the target organism [70]. As this seems to be the case with our choice of a Pseudomonad representative, we believe that the results can be considered meaningful. Nevertheless, this technique has to be tested with the pathogenic variant *P. aeruginosa* according to the standard DIN EN ISO 14729 for contact lens disinfection equipment prior to routine usage.

The reasons for combination testing can be various with different favorable outcomes, such as increasing the effectiveness or decreasing the dosage while increasing or maintaining the same efficacy to avoid toxicity [46]. Minimizing or slowing down the development of drug resistance can also be a motivation [46]. This study was designed mainly to address the second aspect. Meyer et al. [71] describe the reduction of a concentration/dose to reach the same effect as before when adding a second component as “synergistic potency“, which is useful to apply in applications with side effects, in comparison to “synergistic efficacy“ where the aim is to enhance the final result by use of the same drug-concentrations as before.

As mentioned before a considerable volume of aggressive ingredients is stored in the polymeric material of contact lenses after disinfection [18]. As contact lens solutions are known to have adverse effects on the patient’s eye [19,20,21,22,23,24,25,26], a reduced concentration will decrease the patient’s risk of epithelial damage. Since some frequently used contact lens solutions are already at the limit of efficacy, a reduction of the formulation is often not possible. For several multipurpose solutions, including ReNu Multiplus and OptiFree Express, it was shown, however, that diluting the original concentration in PBS led to higher cell viability and integrin expression [72], using concentrations between 1% and 10%, compared to 100%. Another study that investigated dilutions of three different multipurpose solutions on mouse fibroblasts reported that a 25% dilution of all solutions tested could be considered non-toxic [73]. Therefore, reducing contact lens solution concentrations seems favorable. We tried to achieve this by a combination with visible violet light of 405 nm.

It was already proven that light as a single method is usable to meet the criteria of contact lens disinfection and a prototype of an applicable, as well as commercially suitable, system has been developed [37]. The combination of disinfection solution and visible light might not only overcome the problems of efficacy limitation of disinfectants due to biocompatibility issues; the existence of a second technique may also prevent complete failure of a specific lens care product as happened in 2006 with an outbreak of *Fusarium solani* keratitis in several parts of the world [74]. Maintaining a system with two different disinfection strategies would not so easily lead to complete ineffectiveness. 

Following on from the disinfection properties of visible light irradiation, the possible impact on the material characteristics of contact lenses has to be investigated prior to the consideration of translation into routine usage. It has to be ensured that not only the required microbiological parameters are met, but simultaneously the lens itself would not be influenced detrimentally. Preliminary tests, applying irradiation doses simulating cumulative exposure over monthly use, revealed only slight changes concerning transmission, still well within the limits defined by the standard DIN EN ISO 18369–2 (data not shown). However, differences to this test protocol can occur in routine use due to rubbing of the lens, so it is recommended to perform further tests concerning material compatibility, including examination of mechanical stability.

## 5. Conclusions

The combination of contact lens disinfection solutions with the application of visible light irradiation could provide the same antimicrobial results as commercially available disinfection systems but with much less toxicity. While only some of the combinations with ReNu Multiplus investigated in this study were close to the disinfection effect of the pure commercial disinfection solution, the collectivity of results suggests that with modest increase in concentration or exposure time the same impact as provided by commercial ReNu Multiplus formulation may be reached. Combination with light was especially effective against pseudomonas, against which the effectiveness of ReNu Multiplus alone was problematic. For the hydrogen peroxide solution examined, the combination effect with visible light was so strong that even 5% of AOSept was sufficient to reach the same result as the current 100% formulation. It was also shown that an additional disinfection technique might be advantageous, especially because light inactivation, as well as a combination approach, does not show limited efficacy at high concentrations of bacteria, which is a problem for ReNu Multiplus’s effectiveness. Furthermore, light cannot exceed expiration date or lose potency unnoticed, so that its additional application can cover the role of a double protection. Nevertheless, material properties of the contact lens after irradiation have to be tested before application of this promising method is possible.

## 6. Patents

K. Hoenes, and M. Hessling have filed a German patent application (DE 10 2016 009 175 A1).

## Figures and Tables

**Table 1:** Combination Index values calculated based on Loewe Additivity for log reduction results on agar plates achieved with ReNu Multiplus and 405 nm on *P. fluorescens*.

	Combination	a	b	A	B	CI
	Log	Dose in J/cm^2^	%	Dose in J/cm^2^	%	
10 mW/cm^2^	−0.82	140	5	137.7	24.8	1.2186
	−2.19	140	20	230.6	47.5	1.0278
	−3.26	140	30	303.1	65.3	0.9215
	−3.86	140	40	344.0	75.3	0.9382
20 mW/cm^2^	−1.12	140	5	169.3	29.5	0.9963
	−2.76	140	20	327.8	60.1	0.7600
	−4.09	140	30	457.0	84.9	0.6595
	−4.49	140	40	496.5	92.5	0.7142
40 mW/cm^2^	−0.76	140	5	226.1	28.4	0.7955
	−0.73	140	20	223.1	27.8	1.3460
	−1.29	140	30	282.5	37.0	1.2841
	−2.01	140	40	359.1	51.2	1.1708
